# Pneumomediastinum as a presenting symptom of perforated sigmoid cancer: a case report

**DOI:** 10.4076/1757-1626-2-7356

**Published:** 2009-06-09

**Authors:** Raymond Farah, Nicola Makhoul

**Affiliations:** 1 Internal Medicine B Department, Ziv Medical CenterP.O.B. 1008, Safed 13100Israel; 2 Respiratory Intensive Care UnitWestern Galilee Hospital-P.O.B. 21, Nahariya 22100Israel

## Abstract

We report a rare case of spontaneous pneumomediastinum due to perforation of sigmoid cancer in a patient suffering from Vogt-Koyanagi-Harada syndrome and temporal arteritis, two rare diseases. This patient, who generally receives corticosteroid and methotrexate therapy, was admitted to hospital with vague abdominal and left flank pain, urinary disorders and low grade fever one day prior to admission. Initial evaluation including X-ray and laboratory tests was normal. Several hours later a repeat chest X-ray showed pneumomediastinum. Chest and abdominal Computed Tomgraphy were performed because of worsening abdominal pain, and revealed a perforated sigma due to carcinoma.

## Introduction

Pneumomediastinum (PM) is uncommon, and spontaneous PM is rare. It occurs when air leaks from any part of the lung or airways into the mediastinum. One cause is increased pressure within the lungs or airways that ruptures the air sacs or airways, allowing air to escape into surrounding structures. Such pressure can be caused by excessive coughing, sneezing, vomiting or repeated Valsalva maneuvers (bearing down to increase abdominal pressure, such as during childbirth or defecation). PM may also occur following perforation of the trachea, rapid ascents in altitude, or use of a breathing machine (mechanical ventilation). It also can occur in association with pneumothorax or other diseases [1]. Pneumomediastinum may not be accompanied by any symptoms. Usually, it causes severe chest pain below the sternum (breastbone) that may radiate to the neck or arms. The pain may be worse with breathing or swallowing. Often, no treatment is required as the air is gradually absorbed from the mediastinum. PM preceding/preceded by colonic perforation is a very rare entity, only a few cases have been described medical literature [2].

## Case presentation

**Figure 1.**
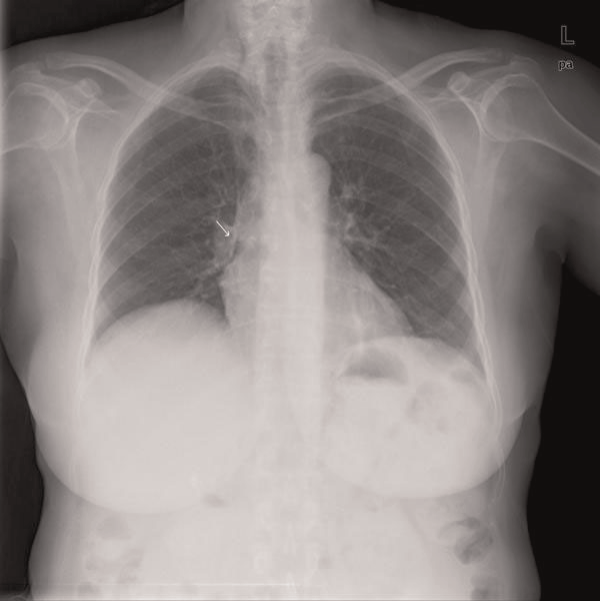
Chest radiogram showed a small initial pneumomediastinum, no pneumothorax is seen (white arrow).

**Figure 2.**
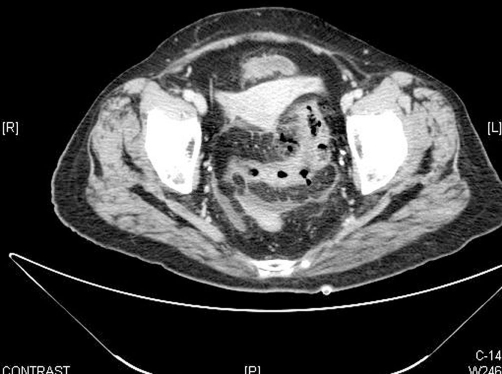
Abdominal CT scan revealed mass and diverticulosis of sigma and perforated cancer with pneumretrooperitoneum.

**Figure 3.**
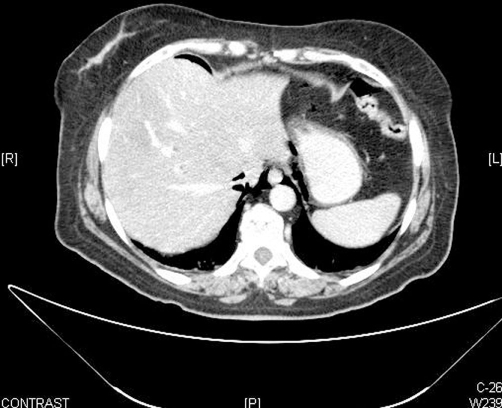
Abdominal CT scan revealed extraperitoneal air from the sigmoid perforation diffusing superiorly through paravertebral retroperitoneal tissue and via the diaphragmatic hiatus into the mediastinum.

A 60-year-old Arab woman with a history of Vogt-Koyanagi-Harada (VKH) syndrome is an uveo encephalomeningitis and temporal arteritis for several years was admitted to our department for evaluation of left flank pain and vague abdominal discomfort, low grade fever, and mild urinary disorders. The patient had been treated with corticosteroids (prednisone 20 mg/day), methotrexate 15 mg/weekly and folic acid 5 mg/day for her rare chronic diseases. On admission, the patient appeared well, complained of mild, constant pain in left flank and diffuse abdomen, without nausea, vomiting or diarrhea. She had no symptoms of dyspnea or chest pain; stools were normal. Physical examination revealed good performance status and no respiratory distress. Body temperature was 37.8Cº, blood pressure 130/65 mmHg, heart rate 108/min, and respiratory rate 15/min. Cardiac examination revealed no gallop, murmurs, or friction rub. Lungs were clear. The abdomen was soft, with mild tenderness on deep palpation especially in the left lower quadrant, but without signs of peritoneal irritation. Bowel sounds were present. Laboratory results upon admission revealed leukocyte count 18000/mm^3^, hemoglobin 12.4 g/dl, and platelets 227000/mm^3^. Blood glucose 160 mg/dl, sodium, potassium and electrolytes were normal. Kidney and liver function tests including amylase were all in the normal range. Urine test was normal. Chest and abdominal X-ray at admission showed normal findings. Chest X-ray was repeated several hours later because of the appearance of mild chest pain and dyspnea, and revealed an initial small PM (Figure 1). On the subsequent day, the patient complained of worsening of abdominal pain, the abdomen was distended with diffuse tenderness and no bowel sounds were detected. Emergency chest and abdominal CT (computed tomography) was conducted and revealed pneumomediastinum and perforated sigma due to carcinoma with pneumoretroperitoneum (Figure 2,3). The patient was rushed to surgery. In surgery, a mass evading the sigmoid colon with a perforation to its mesentery was found. Hartmann's procedure with resection of the sigmoid colon was performed. The histological results of the mass were consistent with carcinoma in situ. The post-operative management included gradual oral feeding, steroids and secondary closure of abdominal wound and oncologic follow-up. Chest X-ray three days later was normal. The patient was discharged on the 9^th^ day post-operative with the diagnosis of perforated cancer of sigma.

## Discussion

SPM is a rare condition. A 2001 review by Chalumeau et al. summarized the available literature [3]. Based on previous studies, they determined a prevalence of SPM ranging from 1 per 800 to 1 per 42,000 pediatric patients presenting to a hospital emergency department. Stack et al. [4] reported a 0.3% incidence of PM in association with asthma presenting to their institution over a 10-year period [4]. The mean age of affected patients was 11 years. No gender differences were observed in their cohort. A study from Nashville, Tennessee, reported the frequency of extra-abdominal gas in a series of patients undergoing laparoscopic esophageal surgery [5]. Forty-seven percent of patients (N = 45) had evidence of extra-abdominal air on chest radiography. Of these, 86% had a PM [5]. PM persisted at least 1 postoperative day in two thirds of these cases. However, no mortality or morbidity was attributable to the presence of PM. The mortality and morbidity associated with PM are generally attributable to underlying disease states. SPM is usually a self-limited condition that rarely produces significant or life-threatening symptoms [1]. The most common morbidities attributable to PM are symptoms such as chest pain, voice change, and cough. Rarely, tension PM may result in decreased cardiac output. Laryngeal compression leading to stridor has been reported. Gas embolism has rarely been reported. In rare cases, SPM may be the first manifestation of an occult perforation in the gastrointestinal tract or other retroperitoneal processes [6,7]. Few reports exist on sigmoid cancer presenting with perforation in which the first sign is PM. Perforation of sigmoid cancer can cause generalized peritonitis and is usually due to microperforation. This complication occurs more frequently in patients with diffuse diverticulosis who are past 50 years of age, and the frequency is increased in patients treated with corticosteroids or other immunosuppressive therapy, as was the case in our patient [2]. Usually the diagnosis of retroperitoneal perforation is difficult because of the lack of signs of peritoneal irritation and the paucity of symptoms, particularly in patients treated with corticosteroids. In our patient, the immunosuppressive therapy caused the impairment of host defense and perforation of cancer. These drugs also were the probable reason for lack of the classic symptoms of perforation and the absence of abscess formation in the retroperitoneum, so the vague abdominal pain in our patient could be related to multiple other causes. In our case, the extraperitoneal air from the sigmoid perforation could escape diffusing superiorly through paravertebral retroperitoneal tissue and via the diaphragmatic hiatus into the mediastinum, and producing pneumomediastinum [8,9]. The CT scan supported the theory of this mechanism. This case illustrates an unusual presentation of perforated sigmoid cancer that was diagnosed by the appearance of SPM as first sign in the simple chest X-ray. When we find PM in elderly patients and in patients treated with steroids, we must consider the rare causes and proceed with specialized testing for correct diagnosis. In our case, surgical intervention may well have prevented life-threatening perforation complications and contamination of the peritoneal cavity and the early diagnosis and resection of the tumor.

## List of abbreviations

CT: Computed tomography
PM: Pneumomediastinum
SPM: Spontaneus pneumomediastinum
VKH: Vogt-Koyanagi-Harda
